# Association of serum 25-hydroxyvitamin D3, fibroblast growth factor-23, and C1q/tumor necrosis factor-related protein-3 with coronary artery calcification in nondialysis chronic kidney disease patients

**DOI:** 10.1080/0886022X.2023.2220412

**Published:** 2023-06-09

**Authors:** Yuanjie Zhu, Zhijuan Hu, Yu Liu, Congcong Qin, Xing Chen, Yanan Shi, Lijun Wang

**Affiliations:** a Graduate School, North China University of Science, Tangshan, China; b Department of Nephrology, Hebei General Hospital, Shijiazhuang, China; c Graduate School, Hebei Medical University, Shijiazhuang, China; d Graduate School, Hebei North University, Zhangjiakou, China

**Keywords:** Nondialysis, CTRP3, FGF23, 25(OH)D3, coronary artery calcification

## Abstract

**Objective:**

To assess serum 25-hydroxyvitamin D3 (25(OH)D3), fibroblast growth factor 23 (FGF23), and C1q/tumor necrosis factor-related protein-3 (CTRP3) levels in nondialysis chronic kidney disease (CKD) patients and their relationship with coronary artery calcification (CAC).

**Methods:**

One hundred and twenty-eight patients diagnosed with CKD were selected and all underwent cardiac computed tomography. CAC was assessed using the Agatston score, and coronary artery calcification score (CACs) >10 was identified as CAC. The differences in serum 25(OH)D3, FGF23, and CTRP3 levels between the CAC and non-CAC groups were analyzed. Their correlation with CACs was assessed by Spearman’s analysis, and logistic regression analysis was used to find risk factors for CAC.

**Results:**

Compared to the non-CAC group, the CAC group was older (64.21 ± 9.68 years), with a higher percentage of hypertension (93.10%) and diabetes (63.80%) and higher levels of serum CTRP3 [1079.20 (644.4–1567.2) ng/mL]. However, there was no significant difference in serum 25(OH)D3 and FGF23 between these two groups. The high level CTRP3 group had a higher prevalence of CAC (61.5%). Logistic regression results showed that age, diabetes, decreased 25(OH)D3 (odds ratio (OR) = 0.95, *p* = .030) and high levels of CTRP3 (OR = 3.19, *p* = .022) were risk factors for CAC in nondialysis CKD patients.

**Conclusions:**

Serum CTRP3 levels progressively increased with the progression of kidney disease, while 25(OH)D3 levels progressively decreased. Decreased 25(OH)D3 and high levels of CTRP3 are associated with CAC in patients with nondialysis CKD.

## Introduction

Chronic kidney disease (CKD) has a prevalence of 10.8% in China [1], posing a serious public health risk. CKD has several complications, including cardiovascular disease (CVD), which is one of the leading causes of death in CKD patients [2]. In addition, 1.4 million deaths from CVD were attributable to impaired kidney function, representing 7.6% of deaths from CVD in 2017 [3]. According to some studies, people with CKD are more likely to experience cardiovascular problems before they need kidney replacement therapy (KRT) [4]. Nondialysis CKD patients are 5–10 times more likely to die from CVD than to progress to dialysis-dependent CKD [5].

Vascular calcification (VC) is one of the manifestations of aging, but diseases such as diabetes, hypertension, and CKD can accelerate this process. Therefore, VC is one of the risk factors for increased CVD morbidity and mortality in CKD patients [6]. Coronary artery calcification (CAC), as a kind of VC, has a strong causal relationship with cardiovascular events and has been increasingly used in clinical research [7]. Once CAC has occurred, it is irreversible. Therefore, it is necessary to find markers to delay the progression of CAC in patients with nondialysis CKD.

C1q/tumor necrosis factor-related protein-3 (CTRP3), also known as CORS26, cartducin, and cartonectin, is a member of the C1q complement-related protein family that was discovered in recent years and is an adipokine with high sequence homology to adiponectin. It is expressed in various tissues, and numerous studies have demonstrated its effects on enhancing insulin sensitivity, regulating metabolism, anti-inflammation, and protecting the cardiovascular system [8]. As an emerging circulating biomarker, CTRP3 has been studied in patients with coronary artery disease, type 2 diabetes, metabolic syndrome, and osteoarthritis [9,10]. However, few studies on CTRP3 have been conducted in patients with CKD.

25-Hydroxyvitamin D3 (25(OH)D3) and fibroblast growth factor 23 (FGF23) are indicators of calcium and phosphate metabolism [11], and their relationship with CAC has been a research hotspot in recent years. However, the conclusions of that aforementioned research are contradictory. Some studies suggested that 25(OH)D3 deficiency and FGF23 are risk factors for VC [12–14], while others have found that they are poorly correlated with calcification [15,16]. Therefore, additional experiments are necessary.

Thus, this study aimed to evaluate the levels of serum 25(OH)D3, FGF23, and CTRP3 in nondialysis CKD patients and their association with CAC.

## Materials and methods

### Patients

Over a 10-month period from June 2021 to April 2022, a total of 177 patients at various CKD stages were recruited from the Nephrology Department of Hebei General Hospital. Forty-nine patients younger than 18 years, on dialysis, with coronary artery disease, primary bone disease, primary hyperuricemia, gout, immune disease, thyroid or parathyroid disease, malignancy, or recent severe infection, surgery, or trauma were excluded. Ultimately, 128 patients were included in this study. This study was approved by the local hospital ethics committee. The approval number is 2023008.

### Clinical and biochemical assessment

The age, sex, height, weight, smoking history, and alcohol use history of all patients were collected. Fasting blood samples were taken, and the Beckman AU680 automatic biochemical analyzer detected the following substances: hemoglobin (Hb), serum albumin (Alb), total bilirubin (TBil), alkaline phosphatase (ALP), urea (BUN), serum creatinine (Scr), uric acid (UA), serum calcium (Ca), serum phosphorus (P), total cholesterol (TC), triglyceride (TG), high-density lipoprotein cholesterol (HDL-C), and superoxide dismutase (SOD). The serum was stored at −80 °C. The CKD-EPI formula published in 2021 was used to estimate glomerular filtration rate (eGFR) [17]. Correction calcium formula: Ca (mmol/L) = serum calcium (mmol/L) + 0.2 × [4-ALB (g/L)/10]. Body mass index (BMI) was calculated based on height and weight. The levels of 25(OH)D3, FGF23, and CTRP3 were measured by human ELISA kits sold on the market (Elabscience, Wuhan, China). The optimal cutoff value of 810.6 ng/mL was calculated according to the receiver operating characteristic (ROC) curve of CTRP3, and all patients were divided into high-level and low-level groups accordingly.

### Coronary artery calcification assessment

All patients underwent cardiac computed tomography (CT). The scanning equipment was the Siemens SOMATOM CT machine. Participants were not administered contrast during the CT scan. The Agatston score was used to evaluate CAC [18]. Calcification was defined as an area of CT peak greater than 130 HU within a 1 mm^2^ area. According to the Rumberger CAC grading method, patients with a score of more than 10 were considered to have CAC [19]. Coronary artery calcification scores (CACs) between 10 and 100 were classified as mild calcification, 100–400 as moderate CAC, and more than 400 as severe CAC.

### Statistical analysis

The effective sample size was determined by the minimum number of observations in the binary outcome, and the number of variables included in the regression analysis was determined according to 10 events per variable. The Kolmogorov–Smirnov test was used to test the normal distributed when the sample size was more than 50, and the Shapiro–Wilk test was used when the sample size was less than 50. Continuous variables with normal distributions are expressed as the mean ± standard deviation (SD), and an independent *t*-test was used for comparisons between the two groups. Non-normal data were expressed as median (interquartile range (IQR)), and the Mann–Whitney *U* test was used for comparisons between groups. Categorical variables were expressed as percentages and compared between groups with the Chi-square test. In the comparison of multiple sets of data, analysis of variance (ANOVA) test was used for data with normal distribution and homoscedasticity, and the Kruskal–Wallis *H* rank test was used for data with a skewed distribution. The Spearman method was used to analyze the correlation between the two indices. Risk factors were analyzed by the binary logistic regression model, and *p* value <.05 was considered statistically significant. All data were analyzed by using IBM Corp. (released 2012, IBM SPSS Statistics for Windows, Version 21.0, IBM Corp., Armonk, NY).

## Results

**Figure 1.**
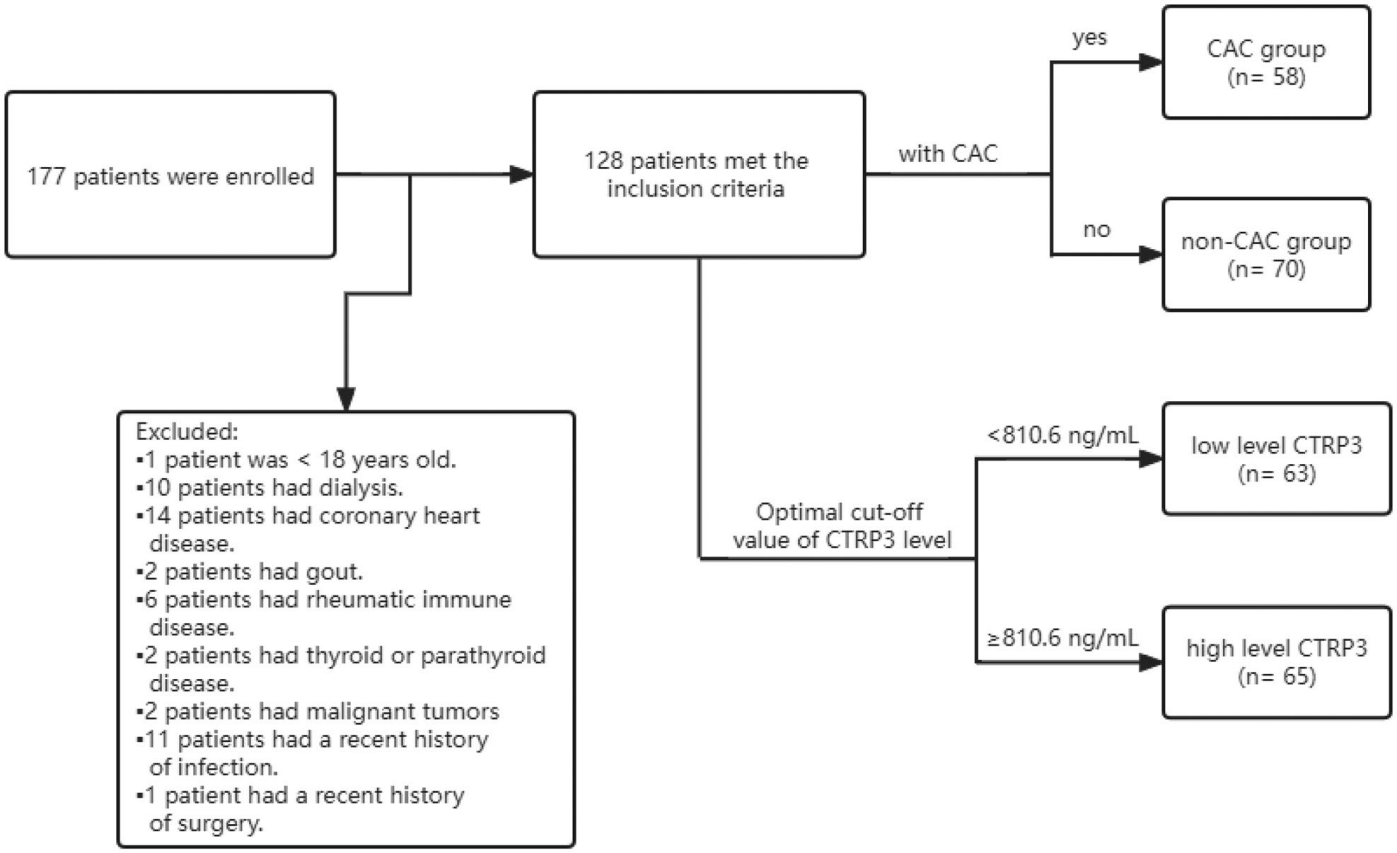
Flow diagram of the study participants.

Figure 1 describes the process of inclusion, exclusion, and grouping of participants in this study. Clinical baseline and biochemical characteristics are shown in Table 1. The average age of the 128 patients in this study was 55.80 ± 14.93 years old, and 85 were male (66.40%); among them, 29 had CKD stages 1 and 2 (22.65%), 30 had CKD stages 3 and 4 (23.44%), and 69 had CKD stage 5 (53.91%). Moreover, 57 patients (44.53%) had diabetes, 106 patients (82.81%) had hypertension, and 54 patients (42.19%) had both diseases. Fifty-eight patients (45.31%) had CAC, among which 35 patients (27.34%) had moderate calcification and 21 patients (16.41%) had severe calcification. The prevalence of CAC was 27.6% in CKD 1–2, 50.0% in CKD 3–4, and 50.7% in CKD 5. Compared with the non-CAC group, the CAC group was older, had higher prevalences of hypertension and diabetes, and had a lower serum Hb level. The level of CTRP3 was significantly higher in the CAC group than in the non-CAC group (*p* = .004). However, there was no significant difference in serum 25(OH)D3 and FGF23 levels between the two groups.

**Table 1.** Baseline characteristics of study participants in CAC and non-CAC groups.

Variable	All patients (*n* = 128)	No-CAC (*n* = 70)	CAC (*n* = 58)	*t*/*Z*/*χ*^2^	*p*
25(OH)D3 (ng/mL)	17.11 (11.23–24.96)	19.43 (12.75–26.97)	16.54 (10.66–21.31)	–1.776	.076
FGF23 (ng/mL)	25.22 (7.11–77.18)	21.45 (3.62–76.37)	33.47 (9.51–79.11)	–0.745	.456
CTRP3 (ng/mL)	818.2 (389.2–372.5)	551.0 (252.5–1169.9)	1079.2 (644.4–1567.2)	–2.846	.004
Male (%)	85 (66.4)	50 (71.4)	35 (60.3)	1.747	.186
Age (years)	55.8 ± 14.9	48.8 ± 14.9	64.2 ± 9.6	–7.006	<.001
Smoking history (%)	31 (24.2)	18 (25.7)	13 (22.4)	0.188	.664
Alcohol use history (%)	21 (16.4)	11 (15.7)	10 (17.2)	0.054	.816
Hypertension (%)	106 (82.8)	52 (74.3)	54 (93.1)	7.891	.005
Diabetes (%)	57 (44.5)	20 (28.6)	37 (63.8)	15.930	<.001
BMI (kg/m^2^)	27.68 (24.83–30.22)	27.21 (23.81–30.11)	27.80 (25.22–30.50)	–0.907	.364
Hemoglobin (g/L) (130–175 g/L)	102.66 ± 28.27	107.70 ± 30.36	96.59 ± 24.43	2.249	.026
Albumin (g/L) (40–55 g/L)	32.55 ± 7.74	34.90 (26.00–39.80)	32.75 (27.38–37.58)	–1.029	.303
Total bilirubin (μmol/L) (0–23 μmol/L)	9.20 (6.83–12.03)	9.64 ± 3.86	9.67 ± 3.29	–0.052	.959
Alkaline phosphatase (U/L) (45–125 U/L)	76.65 (62.93–97.63)	72.35 (62.70–92.45)	80.20 (63.98–100.13)	–1.175	.240
Calcium (mmol/L) (2.11–2.52 mmol/L)	2.25 (2.11–2.34)	2.23 (2.11–2.35)	2.27 (2.11–2.34)	–0.553	.580
Phosphorus (mmol/L) (0.85–1.51 mmol/L)	1.68 ± 0.48	1.65 ± 0.49	1.72 ± 0.47	–0.783	.435
Urea (mmol/L) (3.1–8.0 mmol/L)	17.50 (8.28–28.55)	15.90 (7.25–28.45)	21.25 (10.30–29.08)	–1.307	.191
Serum creatinine (μmol/L) (57–97 μmol/L)	376.2 (125.8–675.5)	314.5 (115.1–752.3)	463.8 (169.8–618.0)	–0.802	.423
Uric acid (μmol/L) (208–428 μmol/L)	420.1 (336.3–486.8)	418.2 (327.5–486.5)	423.8 (361.8–487.6)	–0.534	.594
eGFR (mL/min/1.73 m^2^)	14.00 (7.00–53.20)	18.00 (6.70–72.20)	10.50 (7.00–35.00)	–1.493	.135
Total cholesterol (mmol/L) (3.0–5.7 mmol/L)	4.93 (3.70–6.24)	4.93 (3.93–6.12)	5.04 (3.41–6.48)	–0.203	.839
Triglyceride (mmol/L) (0.1–1.7 mmol/L)	1.48 (0.97–2.08)	1.30 (0.96–2.01)	1.61 (0.99–2.23)	–0.584	.559
HDL-C (mmol/L) (1.16–1.42 mmol/L)	1.08 (0.87–1.33)	1.09 (0.89–1.27)	1.07 (0.85–1.41)	–0.026	.979
SOD (U/mL) (130–215 U/mL)	97.38 ± 34.04	103.70 ± 36.77	89.75 ± 28.94	2.400	.180

25(OH)D3: 25-hydroxyvitamin D3; FGF23: fibroblast growth factor 23; CTRP3: C1q/TNF-related protein-3; BMI: body mass index; eGFR: estimated glomerular filtration rate; HDL-C: high-density lipoprotein cholesterol; SOD: superoxide dismutase.

**Figure 2.**
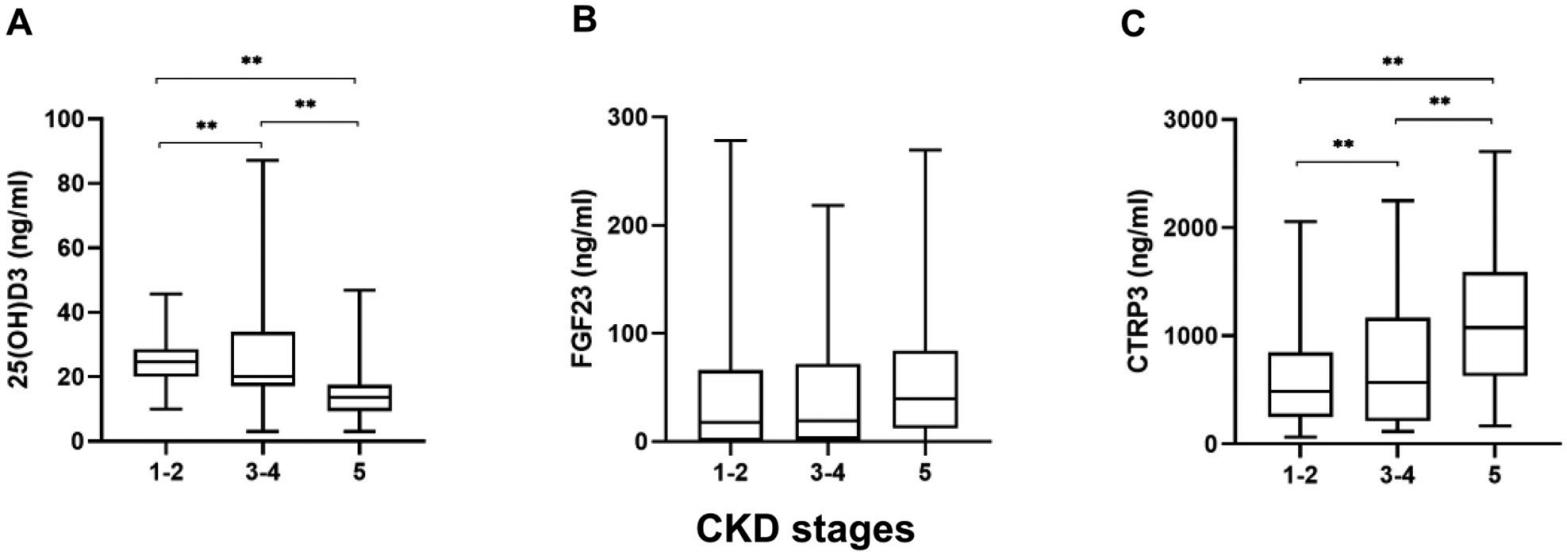
Serum 25(OH)D3, FGF23, and CTRP3 levels at different stages of CKD. (A) Serum 25(OH)D3 levels in CKD stages 1–5, (B) serum FGF23 levels in CKD stages 1–5, and (C) serum CTRP3 levels in CKD stages 1–5. **p < .05.

**Figure 3.**
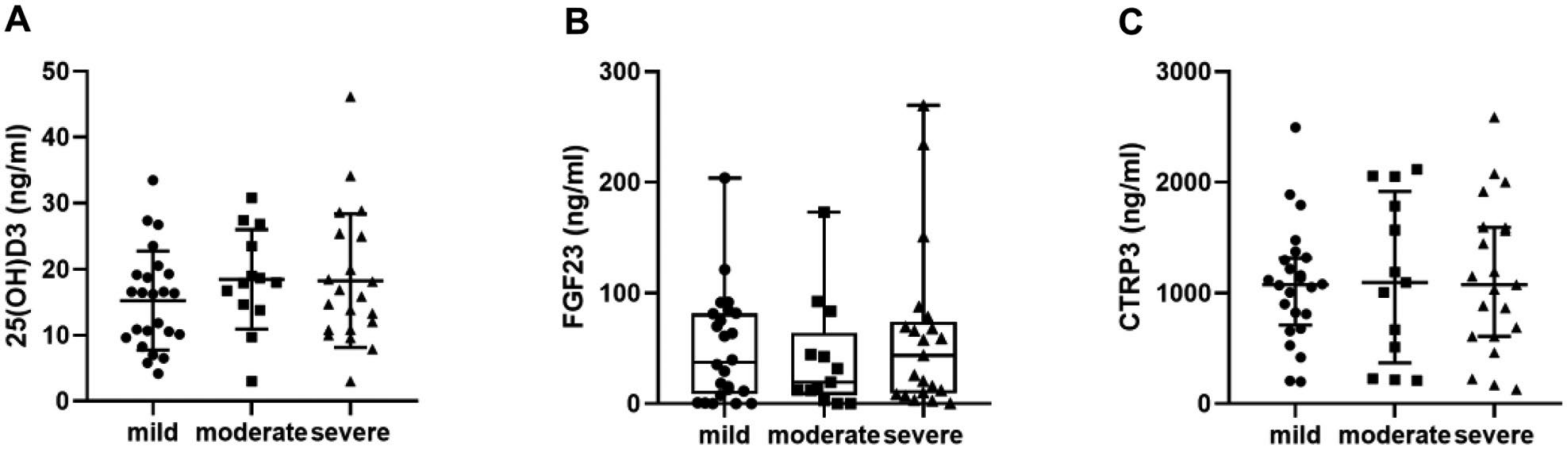
Serum 25(OH)D3, FGF23, and CTRP3 levels in different degrees of CAC. (A) Distribution of serum 25(OH)D3 level in mild, moderate, and severe calcification groups; (B) distribution of serum FGF23 level in mild, moderate, and severe calcification groups; (C) distribution of serum CTRP3 level in mild, moderate, and severe calcification groups.

Table 2 shows the difference between high- and low-level CTRP3 groups. In addition to the higher prevalence of CAC, hypertension, and diabetes, the high CTRP3 group had significantly lower calcium, eGFR, 25(OH)D3, cholesterol, and TG levels and higher phosphorous, urea, and Scr levels (*p* < .05). However, there was no significant difference in BMI or FGF23 between the two groups. With the progression of CKD, the level of serum CTRP3 increased gradually (*H* = 14.827, *p* = .001), and the level of 25(OH)D3 decreased gradually (*H* = 40.834, *p* < .001); in contrast, the level of FGF23 (*H* = 3.904, *p* = .142) did not change significantly (Figure 2). Interestingly, there was no significant difference in serum 25(OH)D3 (*F* = 0.905, *p* = .410), FGF23 (*H* = 0.415, *p* = .813), and CTRP3 (*F* = 0.070, *p* = .933) levels among patients with different degrees of calcification (Figure 3). In nondialysis CKD patients, CTRP3 was positively correlated with ALP, P, BUN, and Scr. It was negatively correlated with Ca, eGFR, and TG. However, there was no correlation between BMI and TC (Table 3). Moreover, the statistically significant indicators in Table 1 and phosphate and eGFR were included in multivariate logistic regression analysis. After adjusting for these factors, age, diabetes, and decreased 25(OH)D3 (odds ratio (OR) = 0.954, *p* = .030) were risk factors for CAC in nondialysis CKD patients. Compared to low levels of CTRP3, CTRP3 ≥ 810.6 ng/mL increased the risk of CAC, and the difference was statistically significant (OR = 3.189, *p* = .022). However, serum FGF23 (*OR* = 0.896, *p* = .863) had no apparent effect on CAC, as shown in Table 4. The effective subgroups for the relationship between 25(OH)D3 and CAC were age ≥60 years, diabetes, and phosphate <1.61 mmol/L. The relationship between CTRP3 and CAC was statistically significant when phosphate was <1.61 mmol/L (Table 5).

**Table 2.** Baseline characteristics of participants at different CTRP3 levels.

Variable	Low level CTRP3 (*n* = 63)	High level CTRP3 (*n* = 65)	*t*/*Z*/*χ*^2^	*p*
25-OH-D3 (ng/mL)	20.57 (14.30–27.85)	14.74 (10.36–19.09)	–3.58	<.001
FGF23 (ng/mL)	18.36 (0.53–61.21)	42.43 (11.83–82.77)	–2.08	.038
CAC (%)	18 (28.60)	40 (61.50)	14.03	<.001
Age (years)	52.81 ± 15.69	58.69 ± 13.65	–2.26	.025
Male (%)	41 (65.10)	44 (67.70)	0.09	.754
Hypertension (%)	46 (73.00)	60 (92.30)	8.36	.004
Diabetes (%)	20 (31.70)	37 (56.90)	8.210	.004
BMI (kg/m^2^)	28.28 (24.61–30.60)	27.43 (24.90–29.65)	–0.97	.331
Alkaline phosphatase (U/L) (45–125 U/L)	70.20 (59.08–84.80)	84.40 (64.30–106.65)	–2.44	.014
Calcium (mmol/L) (2.11–2.52 mmol/L)	2.2 8(2.18–2.37)	2.20 (2.05–2.32)	–2.65	.008
Phosphorus (mmol/L) (0.85–1.51 mmol/L)	1.54 ± 0.42	1.81 ± 0.51	–3.34	.001
Urea (mmol/L) (3.1–8.0 mmol/L)	11.40 (6.80–23.70)	24.70 (14.50–31.95)	–4.12	<.001
Serum creatinine (μmol/L) (57–97 μmol/L)	179.10 (99.80–470.00)	535.00 (318.90–788.00)	–4.09	<.001
Uric acid (μmol/L) (208–428 μmol/L)	424.73 ± 109.09	428.38 ± 128.23	–0.14	.888
eGFR (mL/min/1.73 m^2^)	38.0 (10.00–76.00)	8.0 (6.00–17.50)	–4.06	<.001
Total cholesterol (mmol/L) (3.0–5.7 mmol/L)	5.44 (4.24–6.39)	4.56 (3.32–5.84)	–2.55	.011
Triglyceride (mmol/L) (0.1–1.7 mmol/L)	1.70 (1.08–2.52)	1.20 (0.95–1.95)	–2.38	.017
HDL-C (mmol/L) (1.16–1.42 mmol/L)	1.08 (0.89–1.36)	1.07 (0.82–1.30)	–0.62	.534

25(OH)D3: 25-hydroxyvitamin D3; FGF23: fibroblast growth factor 23; CAC: coronary artery calcification; BMI: body mass index; eGFR: estimated glomerular filtration rate; HDL-C: high-density lipoprotein cholesterol.

**Table 3.** Spearman’s correlation analysis between index and CTRP3.

Variable	*ρ*	*p*
BMI	–0.037	.679
Alkaline phosphatase	0.204	.021
Calcium	–0.237	.007
Phosphorus	0.285	.001
Urea	0.310	<.001
Serum creatinine	0.343	<.001
eGFR	–0.338	<.001
Total cholesterol	–0.163	.066
Triglyceride	–0.257	.003
HDL-C	0.032	.722

BMI: body mass index; eGFR: estimated glomerular filtration rate; HDL-C: high-density lipoprotein cholesterol.

**Table 4.** Binary logistic regression of CAC.

Variable	*B*	OR	95%CI	*p*
Age	0.107	1.113	1.065–1.163	<.001
Diabetes	1.321	3.747	1.428–9.831	.007
25(OH)D3	–0.047	0.954	0.914–0.995	.030
FGF23	–0.110	0.896	0.257–3.118	.863
High level CTRP3	1.160	3.189	1.183–8.596	.022

25(OH)D3: 25-hydroxyvitamin D3; FGF23: fibroblast growth factor 23; CTRP3: C1q/tumor necrosis factor-related protein-3; high level CTRP3: CTRP3 ≥ 810.6 ng/mL.

**Table 5.** Subgroup analysis of risk factors for CAC.

		25(OH)D3		FGF23		CTRP3	
		OR (95%CI)	*p*	OR (95%CI)	*p*	OR (95%CI)	*p*
Age (years)	<60 (*n* = 71)	0.987 (0.923–1.056)	.709	1.002 (0.994–1.011)	.589	3.316 (0.907–12.123)	.070
	≥60 (*n* = 58)	0.938 (0.893–0.986)	.012	0.998 (0.986–1.010)	.757	3.256 (0.887–11.953)	.075
Diabetes	Yes (*n* = 57)	0.944 (0.892–0.998)	.042	1.005 (0.994–1.016)	.399	3.655 (0.989–13.506)	.052
	No (*n* = 72)	0.971 (0.915–1.029)	.321	0.994 (0.981–1.007)	.394	3.333 (0.818–13.577)	.093
Phosphate (mmol/L)	<1.61 (*n* = 63)	0.927 (0.868–0.991)	.025	1.008 (0.996–1.020)	.218	6.575 (1.566–27.600)	.010
	≥1.61 (*n* = 65)	0.980 (0.930–1.033)	.455	0.994 (0.984–1.004)	.229	1.674 (0.463–6.054)	.432

25(OH)D3: 25-hydroxyvitamin D3; FGF23: fibroblast growth factor 23; CTRP3: C1q/tumor necrosis factor-related protein-3.

Age, hypertension, diabetes, phosphate, and eGFR were included in the subgroup analysis.

## Discussion

This study described the levels of circulating 25(OH)D3, FGF23, and CTRP3 in patients with different CKD stages and explored their association with CAC. Compared with the non-CAC group, patients in the CAC group had higher serum CTRP3 levels but no significant difference in FGF23 and 25(OH)D3 levels. People with high CTRP3 levels have a higher rate of CAC. Moreover, increased CTRP3 and decreased 25(OH)D3 were significantly associated with CAC in nondialysis CKD patients.

Zhou et al. found elevated levels of CTRP3 in the serum and abdominal aorta of rats with chronic kidney failure [20]. Yavuz et al. showed that serum CTRP3 levels increased with decreasing GFR in CKD, and this upward trend was more obvious in hemodialysis patients [21]. Additionally, the negative correlation between CTPR3 and GFR still remains in nondialysis patients [22]. The results of this study are consistent with these views. As CKD progresses, serum CTRP3 levels gradually increase. Since the experiment did not design a normal control group but referred to the trend of CTRP3 levels with kidney function changes, it can be speculated that the baseline CTRP3 levels in CKD patients are higher than those in normal people, and this speculation has been confirmed by Yavuz et al. [21]. In particular, there was a positive correlation between CTRP3 and ALP. It is speculated that CTRP3 may directly or indirectly increase the activity of ALP in vascular smooth muscle cells (VSMCs), upregulate the expression of osteogenic marker genes, including runt-related transcription factor 2 (Runx2) [20], and promote the deposition of calcium and formation of calcium nodules. In addition, CTRP3 was positively correlated with phosphate. It is uncertain whether CTRP3 is involved in the complex process of calcium and phosphorus metabolism because serum CTRP3 and phosphate levels gradually increase with decreasing GFR. Further experiments are needed to confirm these results.

Some studies have shown that GFR is closely related to 25(OH)D3 and FGF23. Desjardins et al. found that FGF23 increased with decreasing kidney function [23,24], while 25(OH)D3 levels decreased with increasing CKD stage in predialysis patients [25,26]. Consistent with these findings, serum 25(OH)D3 levels were negatively correlated with kidney function. However, there was no significant difference in serum FGF23 levels between CKD 5 and CKD 1–4 patients in this study, which may be due to the lack of dietary phosphorous intake and drug use. In addition, the normal phosphate baseline level of the total study sample may also be one of the reasons why FGF23 levels did not fluctuate significantly. If these participants were compared with a population with normal kidney function, it is possible that there would be a difference in FGF23 levels. Studies investigating whether serum FGF23 is associated with CAC have shown contradictory results [27]. Srivaths et al. concluded that elevated FGF23 was significantly associated with CAC in pediatric hemodialysis patients [11] but not in adult patients [28]. However, for nondialysis patients, Desjardins et al. believed that FGF23 was independently associated with aortic calcification but had a low correlation with CAC [23]. Moreover, a study of 1501 nondialysis patients showed that baseline FGF23 was not associated with the prevalence and severity of CAC, and the addition of FGF23 to human VSMCs did not promote calcification *in vitro* under normal or high phosphate conditions [15]. Our logistic regression analysis found no correlation between FGF23 and CAC. These results suggest that the relationship between FGF23 and CAC is unstable. For the association between 25(OH)D3 and CAC, several studies have shown that low levels of 25(OH)D3 are linked to greater arterial calcification scores [25] and that patients with higher calcification scores have lower levels of 25(OH)D3 [26]. However, the study of Pillar et al. [16] showed no significant correlation between 25(OH)D3 and CAC, and there was no difference in 25(OH)D3 levels between different calcification degrees. In this research, although 25(OH)D3 deficiency was a risk factor for CAC, the 25(OH)D3 level in the CAC group was not significantly lower than that in the non-CAC group. We speculate that this contradictory result may be because FGF23 inhibits the activity of 1-α-hydroxylase, a key enzyme in vitamin D activation. However, its level did not continue to decrease due to elevated CAC scores. More specifically, there was no correlation between 25(OH)D3 and the severity of calcification [29].

The CAC risk factors identified in our study included higher serum CTRP3 levels, age, diabetes, and decreased 25(OH)D3. Previous studies have shown that in people with normal kidney function, circulating CTRP3 levels are significantly higher in CAD patients than in non-CAD patients and are positively correlated with disease progression [30,31]. Higher serum CTRP3 levels were also observed in patients with CKD combined with CAC. Wang et al. suggested that elevated CTRP3 levels may be a defensive response to CAD [31], and the results of the present study also suggests this claim. In a rat model of chronic kidney failure with CTRP3 overexpression, VC was increased. Zhou et al. suggested that in a high phosphorus environment, CTRP3 could promote VC by promoting β-glycerophosphate-induced osteogenic phenotype transformation of VSMCs, promoting reactive oxygen species (ROS) production in VSMCs and by upregulating Runx2 expression [20]. In this paper, the group with higher CTRP3 also had higher phosphorus levels, which was similar to the above animal experiment results. The phosphate and CTRP3 levels gradually increased as kidney function decreased, but whether they work together to promote calcification remains unclear. In addition, subgroup analysis showed that CTRP3 may also promote CAC at low phosphorus levels. However, this speculation still needs basic research for verification. When we further explored the relationship between CTRP3 and calcification severity, we found no clear trends between CTRP3 levels and different degrees of calcification. If further studies including long-term follow-up of patients with calcification and recalculation of CAC are possible, the progression of coronary calcification may be shown to be associated with the change in CTRP3 level.

In nondialysis CKD patients, this study suggests that 25(OH)D3 and CTRP3 may be involved in the process of CAC through direct or indirect effects. Whether 25(OH)D3 and CTRP3 can be used as biomarkers of cardiovascular events in CKD patients remains to be further studied.

There are still many limitations in this study. First, the sample size was too small, and all samples were from the same region. There might be some selection bias, and the results from various areas and different samples might differ. Second, we lacked information on phosphate intake and medication when collecting patient information, which may have influenced the results. In addition, this study is cross-sectional and cannot assess the cause-effect relationship between CAC and CTRP3. Larger multicenter studies are needed in the future.
